# *Akkermansia muciniphila* Associated with Improved Linear Growth among Young Children, Democratic Republic of the Congo

**DOI:** 10.3201/eid2901.212118

**Published:** 2023-01

**Authors:** Christine Marie George, Alves Birindwa, Shan Li, Camille Williams, Jennifer Kuhl, Elizabeth Thomas, Ruthly François, Amani Sanvura Presence, Bisimwa Rusanga Jean Claude, Patrick Mirindi, Lucien Bisimwa, Jamie Perin, O. Colin Stine

**Affiliations:** Johns Hopkins Bloomberg School of Public Health Department of International Health, Baltimore, Maryland, USA, and Bukavu, Democratic Republic of the Congo (C.M. George, A. Birindwa, C. Williams, J. Kuhl, E. Thomas, R. François, A.S. Presence, B.R. Jean Claude, L. Bisimwa, J. Perin);; University of Maryland School of Medicine Department of Epidemiology and Public Health, Baltimore (S. Li, O.C. Stine);; Food for the Hungry, Washington DC, USA, and Bukavu (P. Mirindi)

**Keywords:** Akkermansia muciniphila, diarrhea, anthropometrics, child growth, prospective cohort studies, bacteria, enteric infections, commensal microbes, pathogenic microbes, Democratic Republic of the Congo, DRC

## Abstract

To investigate the association between enteric pathogens*,* fecal microbes, and child growth, we conducted a prospective cohort study of 236 children <5 years of age in rural eastern Democratic Republic of the Congo. We analyzed baseline fecal specimens by quantitative PCR and measured child height and weight at baseline and growth at a 6-month follow-up. At baseline, 66% (156/236) of children had >3 pathogens in their feces. We observed larger increases in height-for-age-z-scores from baseline to the 6-month follow-up among children with *Akkermansia muciniphila* in their feces (coefficient 0.02 [95% CI 0.0001–0.04]; p = 0.04). Children with *Cryptosporidium* in their feces had larger declines in weight-for-height/length z-scores from baseline to the 6-month follow-up (coefficient –0.03 [95% CI –0.05 to –0.005]; p = 0.02). Our study showed high prevalence of enteric pathogens among this pediatric cohort and suggests *A. muciniphila* can potentially serve as a probiotic to improve child growth.

An estimated 500,000 deaths globally are attributed to diarrheal diseases each year among children <5 years of age (*1*). Enteric pathogens infecting the intestinal tract can cause diarrhea and reduce a child’s ability to absorb nutrients, even when infections are asymptomatic, resulting in malnutrition and impaired growth (*2*,*3*). Globally, in 2021, a total of 149 million children <5 years of age were estimated to be stunted in growth (*4*). Enteric diseases can have long-lasting effects; studies have found that early childhood enteric infections leading to unmet energetic demands for adequate brain development can result in adverse cognitive developmental outcomes later in life (*5*–*7*). In the Democratic Republic of the Congo (DRC), an estimated 45 million diarrheal episodes occur each year, contributing to 10% of deaths among children <5 years of age; 43% of children in this age group are estimated to have stunted growth (*8*–*10*).

A recent study found that the presence of *Akkermansia muciniphila,* a commensal microorganism, in child fecal samples was associated with significantly less diarrhea and greater linear growth measured using height-for-age (HAZ) z-scores (Almeida et al., unpub data). We conducted this cross-sectional study as part of the Global Enteric Multicenter Study (GEMS) conducted in Mali, Kenya, Gambia, and Bangladesh. Additional prospective studies are needed, however, to investigate the association between *A. muciniphila* and child growth. *Lactobacillus* spp. have also been shown protective against enteric infections and associated with healthy gut microbiota composition (*11*–*13*). In a multicountry study, the presence of *L. salivarius* was associated with less *Shigella*-attributed diarrhea (*14*). Laboratory studies have found that *L. salivarius* can improve growth in animals (*15*), but no study has investigated this association in humans.

The Reducing Enteropathy, Undernutrition, and Contamination in the Environment (REDUCE) study focuses on identifying pathways of exposure to fecal pathogens that are significant contributors to diarrheal diseases for young children in the DRC, and on developing and evaluating scalable interventions to reduce fecal contamination from these pathways. Our primary objective in conducting this prospective cohort study was to determine whether the presence and quantity of enteric microorganisms, including *L. salivarius* and *A. muciniphila,* in feces was significantly associated with growth in young children in rural DRC. We hypothesized that the enteric pathogens *Giardia*, *Shigella*, *Cryptosporidium* spp*.*, and *Campylobacter jejuni* would impair child growth by increasing intestinal inflammation and reducing nutrient absorption. Conversely, we hypothesized that *L. salivarius* and *A. muciniphila* would improve child growth by reducing intestinal inflammation and facilitating nutrient absorption.

## Methods

### Study Design

We conducted this prospective cohort study of 236 children <5 years of age in rural Walungu Territory in South Kivu Province, DRC as part of the REDUCE program. The study was part of a larger USAID/Bureau for Humanitarian Assistance–funded Development Food Security Activities (DFSA) awarded with the goal of improving food and nutrition security and economic wellbeing in vulnerable households in South Kivu and Tanganyika provinces in DRC. We enrolled participants during June 2018–January 2019 and conducted 6-month follow-up visits in households during December 2018–August 2019. The number of samples for analysis was determined on the basis of the number of participants with baseline fecal samples and child growth data. We included in the analysis data from all children meeting these criteria who were <5 years of age at follow-up with >5 months of surveillance data from baseline to follow-up. Caregivers were administered a questionnaire at baseline to obtain information on demographic factors. Informed consent was obtained from a parent or guardian of all study participants. Study procedures were approved by the research ethics review committees of the University of Kinshasa (protocol 043-2017) and the Johns Hopkins Bloomberg School of Public Health (protocol 8057).

At baseline, caregivers were provided DNase/RNase-free feces cups and a cooler box with an icepack for collecting and storing feces specimens from children <5 years of age during home visits. Research assistants then transported fecal samples within 6 hours of collection to the microbiology laboratory at the Catholic University of Bukavu in Bukavu, DRC, where samples were stored in liquid nitrogen. Research assistants with training in standardized anthropometry measured children’s weight 1 time and height or length 3 times at baseline and 6-month follow-up. We measured length for children <23 months and height for children 24–59 months of age. We used these measurements to calculate z-scores according to World Health Organization (WHO) child growth standards (*16*): height-for-age (HAZ), weight-for-age (WAZ), and weight-for-height/length z-scores (WHLZ).

### Laboratory Analysis

Fecal samples were transported on dry ice in temperature-controlled shipping containers to the Enteric Microbiology Laboratory at the University of Maryland School of Medicine in Baltimore, Maryland, USA, and stored in a freezer at −80° C until analysis. We isolated DNA from frozen fecal samples using a modified procedure that included bead-beating steps and an adapted QIAGEN QIAamp (https://www.qiagen.com) DNA stool extraction procedure (*17*). We measured concentration of DNA using a Nanodrop spectrophotometer (ThermoFisher Scientific, https://www.thermofisher.com). We analyzed DNA for *Shigella* spp., ETEC, *C. jejuni*, *G. intestinalis*, and *Cryptosporidium* spp. by quantitative PCR (qPCR) using primers published elsewhere (*18*,*19*) and SYBR Green. In addition, we analyzed 2 commensal bacteria, *A*. *muciniphila* (forward primer TCCATCATGAGCCTGTCCGA and reverse primer ACGAGCACCAGAATGATCAG) and *L. salivarius* (forward primer TTATCATTTTAGGCGTCTGGA and reverse primer ATGGGAGACTTGGTTGGATG). We determined gene copies in the specimens by quantification using a standard curve based on dilutions of purified total genomic DNA isolated by QIAGEN column for each 96-well plate (*14*). We combined the DNA concentration, qPCR measurement, and standard curve to estimate the number of gene copies per 100 ng of total fecal DNA. We set >1 copy/100 ng DNA as the cutoff to define the presence of an enteric microorganism, using methods published elsewhere (*20*,*21*).

### Statistical Analysis

To assess the association between enteric microorganisms and measures of child growth, we performed analyses in linear regression models using generalized estimating equations to account for clustering at the household level and to approximate 95% CIs; we recorded changes in HAZ, WAZ, and WHLZ from baseline to the 6-month follow-up as outcomes and presence and quantities of enteric microorganisms as predictors. We adjusted models to account for caregiver formal education (household education), number of persons in the household (household size), household wall type (housing type), breastfeeding (exclusive, any, or none), and animal-source food intake (nutritional status measured using a structured dietary questionnaire on foods consumed in the 24 hours before sampling). We included household education because previous studies had found association between this variable and child growth (*22*,*23*). We included household wall type as a measure of socioeconomic status of the household, which has been associated with child growth (*24*). We included household size as a measure of crowding, which has been associated with food insecurity and delayed growth in young children (*24*–*26*). We included breastfeeding because of studies demonstrating association between this variable and improved child growth and reduced diarrheal diseases (*27*,*28*). We included animal-source food intake because of association between this variable and child growth (*29*,*30*). To assess factors associated with the presence of *A. muciniphila*, we performed analyses on linear regression models using generalized estimating equations to account for clustering at the household level and to approximate 95% CIs, using presence of *A. muciniphila* as the outcome and factors such as age and sex as predictors. We compared children with and without anthropometric data at the 6-month follow-up using a χ^2^ test and performed analyses in SAS version 9.4 software (SAS Institute Inc., https://www.sas.com).

## Results

We obtained baseline fecal samples and anthropometric measurements for 236 children. Median (±SD) baseline age for participants was 2 ±1 years (range 0.08–5.00 years) (Table 1). Girls accounted for 52% (153/236) of participants; 71% (167/236) resided in a household with >1 persons with any level of formal education. Caregivers reported 54% (127/236) of children had any or exclusive breastfeeding in the 24 hours before sampling; 30% (8/27) of children <6 months of age were exclusively breastfed. Other variables among participating children included 69% (163/236) consuming animal-source food in the 24 hours before sampling. For wall materials, 60% (153/236) of participants resided in households with mud walls, 4% (9/236) wood, 5% (12/236) concrete, 6% (13/236) wood and mud, 5% (12/236) biomass, 2% (5/236) brick, and 7% (16/236) wood and concrete.

**Table 1:** Baseline demographic characteristics for participants in study of *Akkermansia muciniphila* association with improved linear growth among young children, Democratic Republic of the Congo*

Characteristic	Value
Children <5 y of age	236
Baseline age, y, median ±SD (range)	1.5 ±1.2 (0.08–4.6)
Sex	
F	122 (52)
M	114 (48)
Household wall type	
Mud walls	153 (66)
Wood walls	9 (4)
Concrete walls	12 (5)
Wood and mud walls	13 (6)
Biomass walls	12 (5)
Brick walls	12 (5)
Wood and concrete walls	5 (2)
Other	16 (7)
Household member with any formal education	167 (71)
Household size, median ±SD (range)	6 ±2.4 (2–17)
Baseline growth measurements, z-score, median ±SD (range)	
Height for age	−2.0 ±1.6 (−5.7 to 5.9)
Weight for height	0.4 ±1.4 (−4.8 to 5.2)
Weight for age	−0.7 ±1.3 (−4.7 to 3.0)

*Values are no. (%) children except as indicated.

We excluded 43 children in our cohort study from analyses because we did not have 6-month follow-up anthropometric measurements for them. We found no significant (p<0.05) differences for any enteric microorganism or demographic factors at baseline between children with or without anthropometric data at 6-month follow-up. At baseline, 95% (224/236) of children had *Giardia*, 54% (127/236) *C. jejuni*, 35% (83/236) *Shigella*, 5% (11/236) *Cryptosporidium*, 70% (166/236) *A. muciniphila*, and 31% (73/236) *L. salivarius* (Table 2). Median copies per 100 ng DNA (range) was 197 (0–2,258,242) for *Giardia,* 1.5 (0–4,724,251) for *C. jejuni*, 0 (0–35,687,711) for *Shigella*, 19 (0–3,290,838) for *Cryptosporidium* spp*.*, 15 (0–17,730,754) for *A. muciniphila*, and 0 (0–1,556) for *L. salivarius*. Median ­+SD pathogens in feces was 3 ±1 (range 0–5). For the number of pathogens, 2% (5/236) of children had zero, 9% (21/236) had one, 23% (54/236) had two, 47% (110/236) had three, 19% had four, and 1 child had five.

**Table 2:** Type and number of enteric pathogens and commensal microbes in feces samples from participants in study of *Akkermansia muciniphila* association with improved linear growth among young children, Democratic Republic of the Congo

Category	No. (%)	Median ±SD (range)
Participants with >1 pathogen in feces, n = 236	73 (89)	3 ±1 (0–5)
Pathogen type		
* Giardia*	224 (95)	197 ±170,042 (0–2,258,242)
* Shigella*	83 (35)	0 ±2,937,024 (0–35,687,711)
* Cryptosporidium*	11 (5)	0 ±214,213 (0–3,290,838)
* Campylobacter jejuni*	127 (54)	1.5 ±487,706 (0–4,724,251)
No. pathogens		
None	10 (4)	
1	58 (25)	
2	118 (50)	
3	49 (20)	
4	1 (1)	
Commensal microbes		
* Akkermansia muciniphila*	166 (70)	15 ± 1,196,734 (0–17,730,754)
* Lactobacillus salivarius*	73 (31)	0 ± 151 (0–1,556)

We observed larger increases in HAZ, 0.34 HAZ coefficient (95% CI 0.2–0.67; p = 0.04), from baseline to 6-month follow-up for children with *A. muciniphila* detected in their feces at baseline compared with those who did not (Table 3). When we included *A. muciniphila* as a continuous outcome (log transformed) in the model, HAZ coefficient was also significantly higher, 0.02 (95% CI 0.0001–0.04; p = 0.04). Children with versus without *Cryptosporidium* spp. in their feces had larger declines in WHLZ, −0.03 WHLZ coefficient (95% CI −0.05 to −0.005; p = 0.02). We observed no other significant associations between enteric pathogens or microbes and child growth. Including caregiver-reported child antibiotic usage in our models did not significantly change our observed associations. Older children had significantly higher *A. muciniphila* in feces (p <0.05) (Appendix Table 1), whereas children <2 years of age had a significantly higher number of enteric pathogens in their feces (p = 0.046) (Appendix Table 2).

**Table 3:** Associations between enteric pathogens and anthropometric measurements for participants in study of *Akkermansia muciniphila* association with improved linear growth among young children, Democratic Republic of the Congo*

Category	Change from baseline to 6-month follow-up, coefficient (95% CI)		
	Height-for-age z-score	Weight-for-height/length z-scores	Weight-for-age z-score
Pathogen or microbe, presence vs. absence			
No. pathogens	0.08 (–0.05 to 0.21)	–0.01 (–0.15 to 0.14)	0.06 (–0.04 to 0.15)
* Giardia*	0.27 (–0.25 to 0.80)	–0.13 (–0.84 to 0.58)	0.02 (–0.36 to 0.41)
* Shigella*	0.01 (–0.30 to 0.33)	0.11 (–0.20 to 0.42)	0.03 (–0.21 to 0.27)
* Cryptosporidium*	0.37 (–0.08 to 0.81)	–0.41 (–0.83 to 0.0008)	0.00 (–0.38 to 0.37)
ETEC	0.11 (–0.19 to 0.42)	–0.26 (–0.60 to 0.09)	0.04 (–0.27 to 0.34)
* Campylobacter jejuni*	0.09 (–0.15 to 0.34)	0.11 (–0.17 to 0.40)	0.16 (–0.05 to 0.37)
* Akkermansia muciniphila*	0.34 (0.02–0.67)	–0.04 (–0.37 to 0.28)	0.23 (–0.01 to 0.47)
* Lactobacillus salivarius*	–0.12 (–0.40 to 0.17)	–0.01 (–0.29 to 0.27)	–0.03 (–0.26 to 0.19)
Pathogen or microbe, log transformed presence vs. absence			
* Giardia*	0.01 (–0.02 to 0.04)	–0.01 (–0.04 to 0.03)	0.0005 (–0.02 to 0.02)
* Shigella*	0.0007 (–0.02 to 0.02)	0.01 (–0.01 to 0.03)	0.01 (–0.01 to 0.02)
* Cryptosporidium*	0.02 (–0.01 to 0.05)	–0.03 (–0.05 to 0.005)	–0.01 (–0.03 to 0.02)
ETEC	–0.01 (–0.03 to 0.01)	0.0004 (–0.02 to 0.02)	0.003 (–0.01 to 0.02)
* Campylobacter jejuni*	0.004 (–0.01 to 0.02)	0.002 (–0.02 to 0.02)	0.01 (–0.01 to 0.02)
* Akkermansia muciniphila*	0.02 (0.001 to 0.04)	–0.01 (–0.03 to 0.01)	0.01 (–0.01 to 0.02)
* Lactobacillus salivarius*	–0.01 (–0.04 to 0.02)	0.00 (–0.03 to 0.03)	–0.001 (–0.02 to 0.02)

*Models adjusted for wall type, household educational level, number of persons in the household, animal source food, and breastfeeding. ETEC, enterotoxigenic *Escherichia coli*.

## Discussion

In this prospective cohort study conducted in rural eastern DRC, we found that *A. muciniphila* was associated with improved linear growth in young children, *Cryptosporidium* was associated with impaired growth, and two thirds of children had a high prevalence (>3) of enteric pathogens in their feces. Children with *Cryptosporidium* in their feces, as measured by WHLZ, grew more poorly as the abundance of the pathogen increased. In contrast, *A. muciniphila* in feces was associated with improved linear growth. This promising finding suggests that *A. muciniphila* may have the potential to serve a probiotic role to help improve growth in young children; however, experimental studies must first be conducted to prove this potential benefit. Children are most susceptible to linear growth faltering during the first 2 years of life (*31*), and effective interventions are urgently needed to improve child health during this critical window of development.

Our finding that *A. muciniphila* was associated with improvements in linear growth is consistent with a recent cross-sectional study among children in GEMS, which found that children who had *A. muciniphila* in their feces had higher HAZ than did children who did not (Almeida et al., unpub. data). Previous studies in adult populations have found *A. muciniphila* more abundant in healthy persons compared with those with inflammatory bowel disease (*32*). *A. muciniphila* resides in the intestinal mucin which may serve as its carbon source (*33*). We hypothesize that *A. muciniphila* impacts the gut mucosal barrier through reducing intestinal inflammation. Our results, however, do not imply causality, and our study is not a substitute for a randomized clinical trial. Low *A. muciniphila* presence may be a marker of pathogenic processes, such as increased intestinal inflammation, contributing to poor child growth, but the microbe itself may not be directly influencing child growth. Mechanistic studies are needed to further investigate our observed association between *A. muciniphila* and child growth.

Findings from human and animal studies suggest that *A. muciniphila* is a highly promising probiotic (*34*). Oral *A. muciniphila* supplementation improved clinical responses to immune checkpoint inhibitors targeting the PD-1/PD-L1 (programmed death-1/programmed death ligand-1) axis in animal studies (*35*), and *A. muciniphila* reduced biomarkers of liver dysfunction and inflammation among persons who were overweight or obese (*36*); however, no studies have investigated its effect on child growth or diarrhea. Rhubarb extract has been shown to promote *A. muciniphila* abundance (*34*) and might therefore serve as a potential natural source of increased *A. muciniphila*. Future mechanistic studies are needed to determine if *A. muciniphila* is associated with decreased enteric inflammation and systemic inflammation. Experimental studies are needed to investigate our observed association between *A. muciniphila* and child growth in other global settings to determine whether this commensal microbe can be used as a potential therapeutic agent to improve child growth.

*Cryptosporidium* is a protozoan parasite that infects the small intestine, resulting in damage to the intestinal epithelium walls, and causes an estimated 44 million diarrheal episodes globally each year, 9 million in sub-Saharan Africa (*37*,*38*). This intestinal damage can reduce nutrient absorption and barrier function and lead to a disorder, named environmental enteropathy, associated with impaired linear growth in young children (*39*,*40*). *Cryptosporidium* is zoonotic in origin and can be spread through cattle and also through fecal–oral transmission (*41*). Consistent with our findings, a recent meta-analysis found that *Cryptosporidium* was associated with declines in WHLZ (*37*). Future studies are needed to determine the predominant *Cryptosporidium* transmission pathways for patients in our study setting in eastern DRC.

Nearly all (98%) children in our study had >1 enteric pathogen in their feces, and 89% had >1. A recent study of hospitalized diarrhea patients (children and adults) at a cholera treatment center in Uvira, South Kivu, DRC, found that 50% of girls and 68% of boys 1–15 years of age had >1 pathogen in their feces, a lower percentage than in our study (*42*). However, this difference is likely because the Uvira study included children older than the children <5 years of age comprising our study cohort; older persons typically have fewer enteric pathogens. The most common enteric pathogen in the Uvira study was *Cryptosporidium*, experienced among 28% of participants.

Among our study’s limitations, we analyzed feces specimens only at baseline, which prevented us from investigating risk factors for subsequent enteric infections or determining the prevalence of enteric infections among our study population over time. Second, we did not perform an in-depth analysis of a larger panel of enteric pathogens from the gut microbiome, which might have provided further information about potential pathways by which enteric microbes affect child growth (*43*). Third, we did not collect information on the HIV status of children. Persons with HIV are at higher risk for *Cryptosporidium* infections (*44*). Fourth, we did not adjust for multiple comparisons; however, all significant findings were in the hypothesized direction. Fifth, we did not have data on diarrhea for all study children; future studies should apply model-derived quantitative cut-points to investigate causes of diarrhea in children (*18*). Finally, our small sample size did not support subgroup analyses by age, which would have been particularly useful for children during the first 2 years of life when they are most susceptible to growth faltering. Future studies should involve larger sample sizes to investigate data by age strata.

Among our study’s strengths, we collected anthropometric data at baseline and 6-month follow-up, using a prospective design that enabled us to assess relationships between enteric infections at baseline and subsequent changes in child growth over time. Second, in addition to enteric pathogens, we included data on commensal microbes, specifically *A. muciniphila* and *L. salivarius*, which could serve as potential therapeutic interventions to promote subsequent child growth. Third, we used qPCR and a bead beating step. qPCR detects enteric microbes at lower concentrations (i.e., qPCR has a higher sensitivity than traditional culture methods [*14**,**45*]). The bead beating step releases more DNA and higher quality DNA than other methods for microbial DNA (*11**,**21*).

In our community-based prospective cohort study, young children had a high burden of enteric pathogens in eastern DRC. We found *Cryptosporidium* in feces was associated with growth faltering, further evidence to support the role of enteric pathogens on child growth in a sub-Saharan Africa setting and highlighting the need for interventions to reduce pediatric exposure to fecal pathogens. Our results also show that *A. muciniphila* was associated with improved linear growth in young children, illustrating the potential of this enteric microbe to serve as a therapeutic intervention for this high-risk population and suggesting pathways for future research globally.

Appendix Additional information for study of *Akkermansia muciniphila* association with improved linear growth among young children, Democratic Republic of the Congo.

## References

[1] Troeger CE, Khalil IA, Blacker BF, Biehl MH, Albertson SB, Zimsen SRM, et al.; GBD 2017 Diarrhoeal Disease Collaborators. Quantifying risks and interventions that have affected the burden of diarrhoea among children younger than 5 years: an analysis of the Global Burden of Disease Study 2017. Lancet Infect Dis. 2020;20:37–59. 10.1016/S1473-3099(19)30401-3 31678029 PMC7340495

[2] Humphrey JH. Child undernutrition, tropical enteropathy, toilets, and handwashing. Lancet. 2009;374:1032–5. 10.1016/S0140-6736(09)60950-8 19766883

[3] George CM, Burrowes V, Perin J, Oldja L, Biswas S, Sack D, et al. Enteric infections in young children are associated with environmental enteropathy and impaired growth. Trop Med Int Health. 2018;23:26–33. 10.1111/tmi.13002 29121442

[4] World Health Organization. Levels and trends in child malnutrition: UNICEF/WHO/The World Bank Group joint child malnutrition estimates: key findings of the 2020 edition [cited 2021 Oct 12]. https://www.who.int/publications/i/item/9789240003576

[5] Prado EL, Dewey KG. Nutrition and brain development in early life. Nutr Rev. 2014;72:267–84. 10.1111/nure.12102 24684384

[6] Berkman DS, Lescano AG, Gilman RH, Lopez SL, Black MM. Effects of stunting, diarrhoeal disease, and parasitic infection during infancy on cognition in late childhood: a follow-up study. Lancet. 2002;359:564–71. 10.1016/S0140-6736(02)07744-9 11867110

[7] Walker SP, Chang SM, Powell CA, Simonoff E, Grantham-McGregor SM. Early childhood stunting is associated with poor psychological functioning in late adolescence and effects are reduced by psychosocial stimulation. J Nutr. 2007;137:2464–9. 10.1093/jn/137.11.2464 17951486

[8] UNICEF. Child and adolescent health: diarrhoeal disease [cited 2021 Oct 12]. https://data.unicef.org/topic/child-health/diarrhoeal-disease</eref>

[9] Diarrhoeal Diseases Collaborators GBD; GBD Diarrhoeal Diseases Collaborators. Estimates of global, regional, and national morbidity, mortality, and aetiologies of diarrhoeal diseases: a systematic analysis for the Global Burden of Disease Study 2015. Lancet Infect Dis. 2017;17:909–48. 10.1016/S1473-3099(17)30276-1 28579426 PMC5589208

[10] Democratic Republic of Congo: demographic and health survey 2013-14: key findings. (English) [cited 2021 Oct 12]. https://dhsprogram.com/pubs/pdf/SR218/SR218.e.pdf

[11] Lindsay B, Oundo J, Hossain MA, Antonio M, Tamboura B, Walker AW, et al. Microbiota that affect risk for shigellosis in children in low-income countries. Emerg Infect Dis. 2015;21:242–50. 10.3201/eid2101.140795 25625766 PMC4313639

[12] Corr SC, Li Y, Riedel CU, O’Toole PW, Hill C, Gahan CG. Bacteriocin production as a mechanism for the antiinfective activity of *Lactobacillus salivarius* UCC118. Proc Natl Acad Sci U S A. 2007;104:7617–21. 10.1073/pnas.0700440104 17456596 PMC1863472

[13] Guandalini S. Probiotics for children with diarrhea: an update. J Clin Gastroenterol. 2008;42(Suppl 2):S53–7. 10.1097/MCG.0b013e3181674087 18520336

[14] Lindsay B, Ochieng JB, Ikumapayi UN, Toure A, Ahmed D, Li S, et al. Quantitative PCR for detection of *Shigella* improves ascertainment of *Shigella* burden in children with moderate-to-severe diarrhea in low-income countries. J Clin Microbiol. 2013;51:1740–6. 10.1128/JCM.02713-12 23536399 PMC3716050

[15] Sayan H, Assavacheep P, Angkanaporn K, Assavacheep A. Effect of *Lactobacillus salivarius* on growth performance, diarrhea incidence, fecal bacterial population and intestinal morphology of suckling pigs challenged with F4+ enterotoxigenic *Escherichia coli.* Asian-Australas J Anim Sci. 2018;31:1308–14. 10.5713/ajas.17.0746 29642683 PMC6043459

[16] de Onis M, Onyango AW. WHO child growth standards. Lancet. 2008;371:204. 10.1016/S0140-6736(08)60131-2 18207015

[17] Black RE, Allen LH, Bhutta ZA, Caulfield LE, de Onis M, Ezzati M, et al.; Maternal and Child Undernutrition Study Group. Maternal and child undernutrition: global and regional exposures and health consequences. Lancet. 2008;371:243–60. 10.1016/S0140-6736(07)61690-0 18207566

[18] Liu J, Platts-Mills JA, Juma J, Kabir F, Nkeze J, Okoi C, et al. Use of quantitative molecular diagnostic methods to identify causes of diarrhoea in children: a reanalysis of the GEMS case-control study. Lancet. 2016;388:1291–301. 10.1016/S0140-6736(16)31529-X 27673470 PMC5471845

[19] Vu DT, Sethabutr O, Von Seidlein L, Tran VT, Do GC, Bui TC, et al. Detection of *Shigella* by a PCR assay targeting the ipaH gene suggests increased prevalence of shigellosis in Nha Trang, Vietnam. J Clin Microbiol. 2004;42:2031–5. 10.1128/JCM.42.5.2031-2035.2004 15131166 PMC404673

[20] Lindsay BR, Chakraborty S, Harro C, Li S, Nataro JP, Sommerfelt H, et al. Quantitative PCR and culture evaluation for enterotoxigenic *Escherichia coli* (*ETEC*) associated diarrhea in volunteers. FEMS Microbiol Lett. 2014;352:25–31. 10.1111/1574-6968.12362 24372618

[21] Pop M, Paulson JN, Chakraborty S, Astrovskaya I, Lindsay BR, Li S, et al. Individual-specific changes in the human gut microbiota after challenge with enterotoxigenic *Escherichia coli* and subsequent ciprofloxacin treatment. BMC Genomics. 2016;17:440. 10.1186/s12864-016-2777-0 27277524 PMC4898365

[22] Boyle MH, Racine Y, Georgiades K, Snelling D, Hong S, Omariba W, et al. The influence of economic development level, household wealth and maternal education on child health in the developing world. Soc Sci Med. 2006;63:2242–54. 10.1016/j.socscimed.2006.04.034 16790308

[23] Wamani H, Astrøm AN, Peterson S, Tumwine JK, Tylleskär T. Predictors of poor anthropometric status among children under 2 years of age in rural Uganda. Public Health Nutr. 2006;9:320–6. 10.1079/PHN2006854 16684383

[24] Tusting LS, Gething PW, Gibson HS, Greenwood B, Knudsen J, Lindsay SW, et al. Housing and child health in sub-Saharan Africa: A cross-sectional analysis. PLoS Med. 2020;17:e1003055. 10.1371/journal.pmed.1003055 32203504 PMC7089421

[25] Haines A, Bruce N, Cairncross S, Davies M, Greenland K, Hiscox A, et al. Promoting health and advancing development through improved housing in low-income settings. J Urban Health. 2013;90:810–31. 10.1007/s11524-012-9773-8 23271143 PMC3795192

[26] Ruiz-Castell M, Muckle G, Dewailly É, Jacobson JL, Jacobson SW, Ayotte P, et al. Household crowding and food insecurity among Inuit families with school-aged children in the Canadian Arctic. Am J Public Health. 2015;105:e122–32. 10.2105/AJPH.2014.302290 25602890 PMC4330833

[27] Onyango AW, Esrey SA, Kramer MS. Continued breastfeeding and child growth in the second year of life: a prospective cohort study in western Kenya. Lancet. 1999;354:2041–5. 10.1016/S0140-6736(99)02168-6 10636370

[28] Lamberti LM, Fischer Walker CL, Noiman A, Victora C, Black RE. Breastfeeding and the risk for diarrhea morbidity and mortality. BMC Public Health. 2011;11(Suppl 3):S15. 10.1186/1471-2458-11-S3-S15 21501432 PMC3231888

[29] Grillenberger M, Neumann CG, Murphy SP, Bwibo NO, Weiss RE, Jiang L, et al. Intake of micronutrients high in animal-source foods is associated with better growth in rural Kenyan school children. Br J Nutr. 2006;95:379–90. 10.1079/BJN20051641 16469157

[30] Kaimila Y, Divala O, Agapova SE, Stephenson KB, Thakwalakwa C, Trehan I, et al. Consumption of animal-source protein is associated with improved height-for-age z scores in rural Malawian children aged 12–36 months. Nutrients. 2019;11:480. 10.3390/nu11020480 30823563 PMC6413013

[31] Victora CG, de Onis M, Hallal PC, Blössner M, Shrimpton R. Worldwide timing of growth faltering: revisiting implications for interventions. Pediatrics. 2010;125:e473–80. 10.1542/peds.2009-1519 20156903

[32] Derrien M, Belzer C, de Vos WM. *Akkermansia muciniphila* and its role in regulating host functions. Microb Pathog. 2017;106:171–81. 10.1016/j.micpath.2016.02.005 26875998

[33] Derrien M, Vaughan EE, Plugge CM, de Vos WM. *Akkermansia muciniphila* gen. nov., sp. nov., a human intestinal mucin-degrading bacterium. Int J Syst Evol Microbiol. 2004;54:1469–76. 10.1099/ijs.0.02873-0 15388697

[34] Zhou K. Strategies to promote abundance of *Akkermansia muciniphila*, an emerging probiotics in the gut, evidence from dietary intervention studies. J Funct Foods. 2017;33:194–201. 10.1016/j.jff.2017.03.045 30416539 PMC6223323

[35] Routy B, Le Chatelier E, Derosa L, Duong CPM, Alou MT, Daillère R, et al. Gut microbiome influences efficacy of PD-1-based immunotherapy against epithelial tumors. Science. 2018;359:91–7. 10.1126/science.aan3706 29097494

[36] Depommier C, Everard A, Druart C, Plovier H, Van Hul M, Vieira-Silva S, et al. Supplementation with *Akkermansia muciniphila* in overweight and obese human volunteers: a proof-of-concept exploratory study. Nat Med. 2019;25:1096–103. 10.1038/s41591-019-0495-2 31263284 PMC6699990

[37] Khalil IA, Troeger C, Rao PC, Blacker BF, Brown A, Brewer TG, et al. Morbidity, mortality, and long-term consequences associated with diarrhoea from *Cryptosporidium* infection in children younger than 5 years: a meta-analyses study. Lancet Glob Health. 2018;6:e758–68. 10.1016/S2214-109X(18)30283-3 29903377 PMC6005120

[38] Kirkpatrick BD, Daniels MM, Jean SS, Pape JW, Karp C, Littenberg B, et al. Cryptosporidiosis stimulates an inflammatory intestinal response in malnourished Haitian children. J Infect Dis. 2002;186:94–101. 10.1086/341296 12089667

[39] Bouzid M, Hunter PR, Chalmers RM, Tyler KM. *Cryptosporidium* pathogenicity and virulence. Clin Microbiol Rev. 2013;26:115–34. 10.1128/CMR.00076-12 23297262 PMC3553671

[40] Kosek M, Haque R, Lima A, Babji S, Shrestha S, Qureshi S, et al. Fecal markers of intestinal inflammation and permeability associated with the subsequent acquisition of linear growth deficits in infants. Am J Trop Med Hyg. 2013;88:390–6. 10.4269/ajtmh.2012.12-0549 23185075 PMC3583335

[41] Xiao L, Feng Y. Zoonotic cryptosporidiosis. FEMS Immunol Med Microbiol. 2008;52:309–23. 10.1111/j.1574-695X.2008.00377.x 18205803

[42] Williams C, Cumming O, Grignard L, Rumedeka BB, Saidi JM, Grint D, et al. Prevalence and diversity of enteric pathogens among cholera treatment centre patients with acute diarrhea in Uvira, Democratic Republic of Congo. BMC Infect Dis. 2020;20:741. 10.1186/s12879-020-05454-0 33036564 PMC7547509

[43] Bagamian KH, Anderson JD IV, Muhib F, Cumming O, Laytner LA, Wierzba TF, et al. Heterogeneity in enterotoxigenic *Escherichia coli* and shigella infections in children under 5 years of age from 11 African countries: a subnational approach quantifying risk, mortality, morbidity, and stunting. Lancet Glob Health. 2020;8:e101–12. 10.1016/S2214-109X(19)30456-5 31734154 PMC7024994

[44] Pieniazek NJ, Bornay-Llinares FJ, Slemenda SB, da Silva AJ, Moura IN, Arrowood MJ, et al. New cryptosporidium genotypes in HIV-infected persons. Emerg Infect Dis. 1999;5:444–9. 10.3201/eid0503.990318 10341184 PMC2640774

[45] Platts-Mills JA, Babji S, Bodhidatta L, Gratz J, Haque R, Havt A, et al.; MAL-ED Network Investigators. Pathogen-specific burdens of community diarrhoea in developing countries: a multisite birth cohort study (MAL-ED). Lancet Glob Health. 2015;3:e564–75. 10.1016/S2214-109X(15)00151-5 26202075 PMC7328884

