# Supplementary material for: Akkermansia muciniphila Associated with Improved Linear Growth among Young Children, Democratic Republic of the Congo
Source: Emerg Infect Dis. 2023 Jan;29(1):81–8. doi: 10.3201/eid2901.212118 (PMC9796213; doi:10.3201/eid2901.212118)
Supplement: Appendix — Additional information for study of Akkermansia muciniphila association with improved linear growth among young children, Democratic Republic of the Congo. [file 21-2118-Techapp-s1.pdf]

Article DOI: https://doi.org/10.3201/eid2811.212118

Akkermansia muciniphila Associated with Improved Linear Growth among Young Children, Democratic Republic of the Congo

Appendix

Appendix Table 1. Factors associated with *Akkermansia muciniphila* presence in feces of children <5 years of age (N = 221)

Variable	No *A. muciniphila* in feces	*A. muciniphila* in feces	p-value
Baseline age, mo			
Median ± SD (min–max)	20 ± 13 (5–53)	25 ± 14 (2–54)	0.01
<24, %	36	64	0.01
24–60, %	18	81	
Sex			
F, %	31	69	0.60
M, %	28	72	
Anthropometric measurements, median ± SD (min–max)			
Height-for-age z-scores	–2.1 ± 1.44 (–5.2 to –1.2)	–1.9 ± 1.7 (–5.7 to –5.9)	0.63
Weight-for-height/length z-scores	0.44 ± 1.5 (–4.8 to 5.2)	0.37 ± 1.5 (–4.7 to 3.2)	0.11
Weight-for-age z-scores	–0.82 ± 1.3 (–3.7 to 2.2)	–0.70 ± 1.3 (–4.7 to 3.0)	0.21
Ingested some animal source food <u><</u>24 h before sampling, %	70	69	0.98
Enteric pathogens, %			
Giardia	90	97	0.08
Shigella	24	40	0.05
Cryptosporidium	3	5	0.60
ETEC	76	88	0.08
Campylobacter jejuni	43	58	0.07

Appendix Table 2. Relationship between age and enteric pathogens in feces of children <5 years of age (N = 221)

Variable	<2 y	2–4 y	p-value
Number of pathogens in feces			
Median ± SD (min–max)	3 ± 1 (0–5)	3 ± 0.9 (0–5)	
0, %	3	1	0.046
1–2, %	30	34	
≥3, %	67	64	
